# Age and sex impact plasma NFL and t-Tau trajectories in individuals with subjective memory complaints: a 3-year follow-up study

**DOI:** 10.1186/s13195-020-00704-4

**Published:** 2020-11-12

**Authors:** Filippo Baldacci, Simone Lista, Maria Laura Manca, Patrizia A. Chiesa, Enrica Cavedo, Pablo Lemercier, Henrik Zetterberg, Kaj Blennow, Marie-Odile Habert, Marie Claude Potier, Bruno Dubois, Andrea Vergallo, Harald Hampel, the INSIGHT-preAD study group, Hovagim Bakardjian, Habib Benali, Hugo Bertin, Joel Bonheur, Laurie Boukadida, Nadia Boukerrou, Enrica Cavedo, Patrizia Chiesa, Olivier Colliot, Bruno Dubois, Marion Dubois, Stéphane Epelbaum, Geoffroy Gagliardi, Remy Genthon, Marie-Odile Habert, Harald Hampel, Marion Houot, Aurélie Kas, Foudil Lamari, Marcel Levy, Simone Lista, Christiane Metzinger, Fanny Mochel, Francis Nyasse, Catherine Poisson, Marie-Claude Potier, Marie Revillon, Antonio Santos, Katia Santos Andrade, Marine Sole, Mohmed Surtee, Michel Thiebaut de Schotten, Andrea Vergallo, Nadjia Younsi, for the Alzheimer Precision Medicine Initiative (APMI), Mohammad Afshar, Lisi Flores Aguilar, Leyla Akman-Anderson, Joaquín Arenas, Jesús Ávila, Claudio Babiloni, Filippo Baldacci, Richard Batrla, Norbert Benda, Keith L. Black, Arun L. W. Bokde, Ubaldo Bonuccelli, Karl Broich, Francesco Cacciola, Filippo Caraci, Giuseppe Caruso, Juan Castrillo, Enrica Cavedo, Roberto Ceravolo, Patrizia A. Chiesa, Massimo Corbo, Jean-Christophe Corvol, Augusto Claudio, Jeffrey L. Cummings, Herman Depypere, Bruno Dubois, Andrea Duggento, Enzo Emanuele, Valentina Escott-Price, Howard Federoff, Maria Teresa Ferretti, Massimo Fiandaca, Richard A. Frank, Francesco Garaci, Hugo Geerts, Ezio Giacobini, Filippo S. Giorgi, Edward J. Goetzl, Manuela Graziani, Marion Haberkamp, Marie-Odile Habert, Britta Hänisch, Harald Hampel, Karl Herholz, Felix Hernandez, Bruno P. Imbimbo, Dimitrios Kapogiannis, Eric Karran, Steven J. Kiddle, Seung H. Kim, Yosef Koronyo, Maya Koronyo-Hamaoui, Todd Langevin, Stéphane Lehéricy, Pablo Lemercier, Simone Lista, Francisco Llavero, Jean Lorenceau, Alejandro Lucía, Dalila Mango, Mark Mapstone, Christian Neri, Robert Nisticò, Sid E. O’Bryant, Giovanni Palermo, George Perry, Craig Ritchie, Simone Rossi, Amira Saidi, Emiliano Santarnecchi, Lon S. Schneider, Olaf Sporns, Nicola Toschi, Pedro L. Valenzuela, Bruno Vellas, Steven R. Verdooner, Andrea Vergallo, Nicolas Villain, Kelly Virecoulon Giudici, Mark Watling, Lindsay A. Welikovitch, Janet Woodcock, Erfan Younesi, José L. Zugaza

**Affiliations:** 1 Sorbonne University, GRC n° 21, Alzheimer Precision Medicine (APM), AP-HP, Pitié-Salpêtrière Hospital, Boulevard de l’hôpital, F-75013 Paris, France; 2 Department of Clinical and Experimental Medicine, University of Pisa, Via Roma 67, 56125 Pisa, Italy; 3 Brain & Spine Institute (ICM), INSERM U 1127, CNRS UMR 7225, Boulevard de l’hôpital, F-75013 Paris, France; 4 Department of Neurology, Pitié-Salpêtrière Hospital, AP-HP, Boulevard de l’hôpital, Institute of Memory and Alzheimer’s Disease (IM2A), F-75013 Paris, France; 5 Department of Mathematics, University of Pisa, Pisa, Italy; 6 Qynapse, Paris, France; 7 Institute of Neuroscience and Physiology, Department of Psychiatry and Neurochemistry, The Sahlgrenska Academy at the University of Gothenburg, Mölndal, Sweden; 8 Clinical Neurochemistry Laboratory, Sahlgrenska University Hospital, Mölndal, Sweden; 9 Department of Molecular Neuroscience, UCL Institute of Neurology, Queen Square, London, UK; 10 UK Dementia Research Institute, London, UK; 11 Laboratoire d’Imagerie Biomédicale, Sorbonne University, CNRS, INSERM, F-75013 Paris, France; 12 Centre pour l’Acquisition et le Traitement des Images (www.cati-neuroimaging.com), Paris, France; 13 AP-HP, Hôpital Pitié-Salpêtrière, Département de Médecine Nucléaire, F-75013 Paris, France; 14 ICM Institut du Cerveau et de la Moelle épinière, CNRS UMR7225, INSERM U1127, UPMC, Hôpital de la Pitié-Salpêtrière, 47 Bd de l’Hôpital, F-75013 Paris, France

**Keywords:** Neurofilament light chain, Tau, Alzheimer’s disease, Subjective memory complainers, Mild cognitive impairment, Biomarkers

## Abstract

**Background:**

Plasma neurofilament light (NFL) and total Tau (t-Tau) proteins are candidate biomarkers for early stages of Alzheimer’s disease (AD). The impact of biological factors on their plasma concentrations in individuals with subjective memory complaints (SMC) has been poorly explored. We longitudinally investigate the effect of sex, age, *APOE ε4* allele, comorbidities, brain amyloid-β (Aβ) burden, and cognitive scores on plasma NFL and t-Tau concentrations in cognitively healthy individuals with SMC, a condition associated with AD development.

**Methods:**

Three hundred sixteen and 79 individuals, respectively, have baseline and three-time point assessments (at baseline, 1-year, and 3-year follow-up) of the two biomarkers. Plasma biomarkers were measured with an ultrasensitive assay in a mono-center cohort (INSIGHT-preAD study).

**Results:**

We show an effect of age on plasma NFL, with women having a higher increase of plasma t-Tau concentrations compared to men, over time. The *APOE ε4* allele does not affect the biomarker concentrations while plasma vitamin B12 deficiency is associated with higher plasma t-Tau concentrations. Both biomarkers are correlated and increase over time. Baseline NFL is related to the rate of Aβ deposition at 2-year follow-up in the left-posterior cingulate and the inferior parietal gyri. Baseline plasma NFL and the rate of change of plasma t-Tau are inversely associated with cognitive score.

**Conclusion:**

We find that plasma NFL and t-Tau longitudinal trajectories are affected by age and female sex, respectively, in SMC individuals. Exploring the influence of biological variables on AD biomarkers is crucial for their clinical validation in blood.

## Introduction

Plasma neurofilament light (NFL) chain and total Tau (t-Tau) proteins are promising biomarkers of Alzheimer’s disease (AD). They show good/fair diagnostic accuracy to distinguish cognitively healthy individuals from AD patients, when ultrasensitive technologies of measurement are used [1–7]. Both proteins are involved in the physiological processes of neuronal integrity [8, 9]. Both plasma t-Tau and NFL are associated with worsening rate of cognitive performance [3, 10–13], cerebral atrophy [2, 3, 12–14], and hypometabolism [2, 3, 12] in prodromal and dementia phases of AD. Only a few studies investigated these biomarkers, either separately [15–18] or in combination [3], during the preclinical stages of sporadic AD. Even less data are available on the effect of key biological factors—such as age, sex, and *APOE ε4* allele—on plasma NFL and t-Tau concentrations. At our knowledge, there are no studies investigating the potential contribution of comorbidities in affecting either plasma NFL or t-Tau concentrations, and no evidence on the impact of these key biological factors on plasma NFL and t-Tau concentrations in populations with SMC, a condition associated with AD development.

Our primary aims are to investigate, in a cohort of cognitively intact individuals with subjective memory complaints (SMC), the dynamic trajectories of plasma NFL and t-Tau concentrations and the impact of sex, age, and *APOE* genotype. Secondary aims are (1) to identify, at baseline, the variables maximally contributing to group separation of individuals above or below the third quartile for baseline NFL and t-Tau concentrations, respectively; (2) to investigate the association of the two plasma biomarkers, at baseline, with baseline global and regional brain amyloid-β (Aβ) deposition, Aβ changes deposition, baseline Mini-Mental State Examination (MMSE) score, Free and Cued Selective Rating Test (FCSRT), the concentrations of the corresponding cerebrospinal fluid (CSF) biomarkers (CSF NFL and CSF t-Tau), and between each other; and (3) to explore the association of NFL and t-Tau changes with MMSE and FCSRT modifications.

## Materials and methods

We performed a longitudinal investigation in 316 SMC participants of the French monocentric “INveStIGation of AlzHeimer’s PredicTors in Subjective Memory Complainers” (INSIGHT-preAD) cohort (Pitié-Salpêtrière University Hospital, Paris) [19]. All participants underwent two Aβ-positron emission tomography (Aβ-PET) scans, at baseline and at 2-year follow-up. A subset of 40 individuals received a baseline lumbar puncture. The concentrations of plasma NFL and t-Tau were measured at three time points using an ultrasensitive technology (*N* = 79 at baseline, 1-year, and 3-year follow-up).

### Study participants

We designed a large-scale mono-centric research program using a cohort of SMC recruited from the INSIGHT-preAD study, a French academic university-based cohort [19] which is part of the Alzheimer Precision Medicine Initiative (APMI) and its Cohort Program (APMI-CP) [20–23]. Participants were enrolled between May 25, 2013, and January 20, 2015, at the Institute of Memory and Alzheimer’s disease (*Institut de la Mémoire et de la Maladie d’Alzheimer, IM2A*) at the Pitié-Salpêtrière University Hospital in Paris, France [19]. The study was conducted in accordance with the tenets of the Declaration of Helsinki of 1975 and approved by the local Institutional Review Board at the participating center. All participants gave written informed consent for use of their clinical data for research purposes.

### PET data acquisition and processing

All Florbetapir-PET scans were acquired in a single session on a Philips Gemini GXL CT-PET scanner 50 (± 5) min after injection of approximately 370 MBq (333–407 MBq) of Florbetapir. Images acquisition, reconstruction including correction algorithms and reallination, averaging, and quality check were performed by the CATI team (*Centre d’Acquisition et Traitement des Images*) (http://cati-neuroimaging.com) [19, 24, 25] and were calculated for each of 12 cortical regions of interest (right and left posterior cingulate, right and left anterior cingulate, right and left superior frontal, right and left inferior parietal, right and left precuneus, right and left middle temporal cortices), as well as the global average standard uptake value ratio (SUVR).

### CSF sampling and biomarkers assessment

CSF sampling was performed by lumbar puncture in a subsample of 40 individuals. All CSF samples were collected in polypropylene tubes, centrifuged at 1000 g for 10 min at + 4 °C. The collected supernatant was stored at − 80 °C for pending biochemical analysis. The immunoassays for CSF core biomarkers are reported in previous studies [26, 27].

### Blood sampling and collection tube storage

Ten (10) mL of venous blood were collected in one BD Vacutainer® spray-coated K2 tube, which was employed for all subsequent immunological analyses. Blood samples were taken in the morning, after a 12-h fast, handled in a standardized way, and centrifuged for 15 min at 2000 G-force at + 4 °C. Per sample, plasma fraction was collected, homogenized, aliquoted into multiple 0.5 mL cryovial-sterilized tubes, and finally stored at − 80°Cwithin 2 h from collection.

### Immunoassays for plasma biomarkers

All analyses of plasma t-Tau and NFL concentrations were performed at the Clinical Neurochemistry Laboratory, Sahlgrenska University Hospital, Sweden [28–30]. In particular, a volume of 0.5 mL of plasma for each individual was required for performing the analyses.

Plasma t-Tau concentrations were measured using the Human Total Tau 2.0 kit on the ultrasensitive single molecule arrays (Simoa) platform (Quanterix, Lexington, MA), according to the manufacturer instructions.

For plasma t-Tau, both repeatability and intermediate precision were 12.2% for an internal QC plasma sample with a concentration of 1.9 pg/mL [2, 30]. The t-Tau assay was originally developed in a collaboration between the Clinical Neurochemistry Laboratory and Quanterix [31]. However, the commercially available assay was built on this work but with another set of antibodies.

Plasma NFL concentrations were measured using the ultrasensitive Simoa technology, according to the manufacturer instructions. Repeatability was 9.6% and 10.6% and intermediate precision was 14.6% and 11.6% for two internal QC plasma samples with concentrations of 12.9 pg/mL and 107 pg/mL [28–30]. The NFL assay was originally developed by the Clinical Neurochemistry Laboratory and then commercialized by Quanterix [32].

All samples were analyzed on one occasion using one batch of reagents by board-certified laboratory technicians who were blinded to clinical data.

### Statistical analysis

Statistical analysis was performed using IBM-SPSS® Statistics, Version 20, and Addinsoft, XLSTAT Statistical and Data Analysis Solution (2019), Long Island, NY, USA, statistical packages for Mac OS X.

Kolmogorov-Smirnov test was applied to check for normality. Gaussian distributed values were expressed as mean and standard deviation (SD) or standard error (SE); otherwise, median and interquartile range (IR) were used for quantitative variables, while categorical data were expressed as frequency.

To evaluate the impact of age, sex, and *APOE ε4* carrier status on NFL and t-Tau evolution in a 3-year follow-up, respectively, we conducted two independent linear mixed-effects models (LMM) on the whole sample [33]. Age, sex, and *APOE ε4* carrier status were included as fixed effects in the model and each individual as random effect. We also included interaction between age and sex, age and *APOE ε4* carrier status, and sex and *APOE ε4* carrier status. Type III likelihood ratio tests were used to test each fixed effect and interaction. The statistical models used were verified for normal distribution of residuals, random effects, and homoscedasticity of residuals. Subsequently, the analysis was repeated after stratifying the sample into amyloid-PET-negative and positive individuals.

On the whole sample, cross-sectional data were also elaborated to identify the variables maximally contributing to group separation of individuals, based on the following outcome variables Y: baseline plasma NFL and baseline plasma t-Tau. To this end, two partial least square (PLS) models were generated, and the variables with Variable Importance in Projection (VIPs, expressing a measure of a variable’s relevance in the model) greater than 1.50 were considered significant for separation of the sample [34, 35]. In the first model, Y was defined by placing NFL = 1 whether NFL concentrations were above or below the third quartile, and 0 if below. Input variables were sex, age, *APOE ε4* carrier status, arterial hypertension, (HTA), atrial fibrillation, heart disease, dyslipidemia, diabetes, obstructive sleep apnea syndrome (OSAS), head trauma, mood disorders, vitamin B12 deficiency, body mass index (BMI), global SUVR, MMSE and FCSRT at baseline, and baseline plasma t-Tau. In the second model, input variables were the same, as previously described, but the outcome variable Y was t-Tau, categorized as 1 if t-Tau concentrations were upper than the third quartile, and 0 otherwise.

The associations of the two plasma biomarkers at baseline with baseline global and regional brain Aβ deposition, Δ Aβ deposition, baseline MMSE score, FCSRT, the corresponding CSF biomarkers, and between each other were evaluated by Spearman correlation testing. If the relationship was significant, a subsequent stepwise forward regression was performed. Spearman test was used to explore the association of Δ NFL and Δ t-Tau with Δ MMSE and Δ FCSRT (cognitive measures). Δ value has been defined as the difference between the baseline and the 3-year follow-up value, excluding for Aβ deposition where we considered 2-year follow-up value, because we had the baseline data available and after 2 years.

Variables with skewed distribution were log-transformed for use in all parametric analyses. *P* values < 0.05 were considered significant in all statistical elaboration.

## Results

**Fig. 1:**
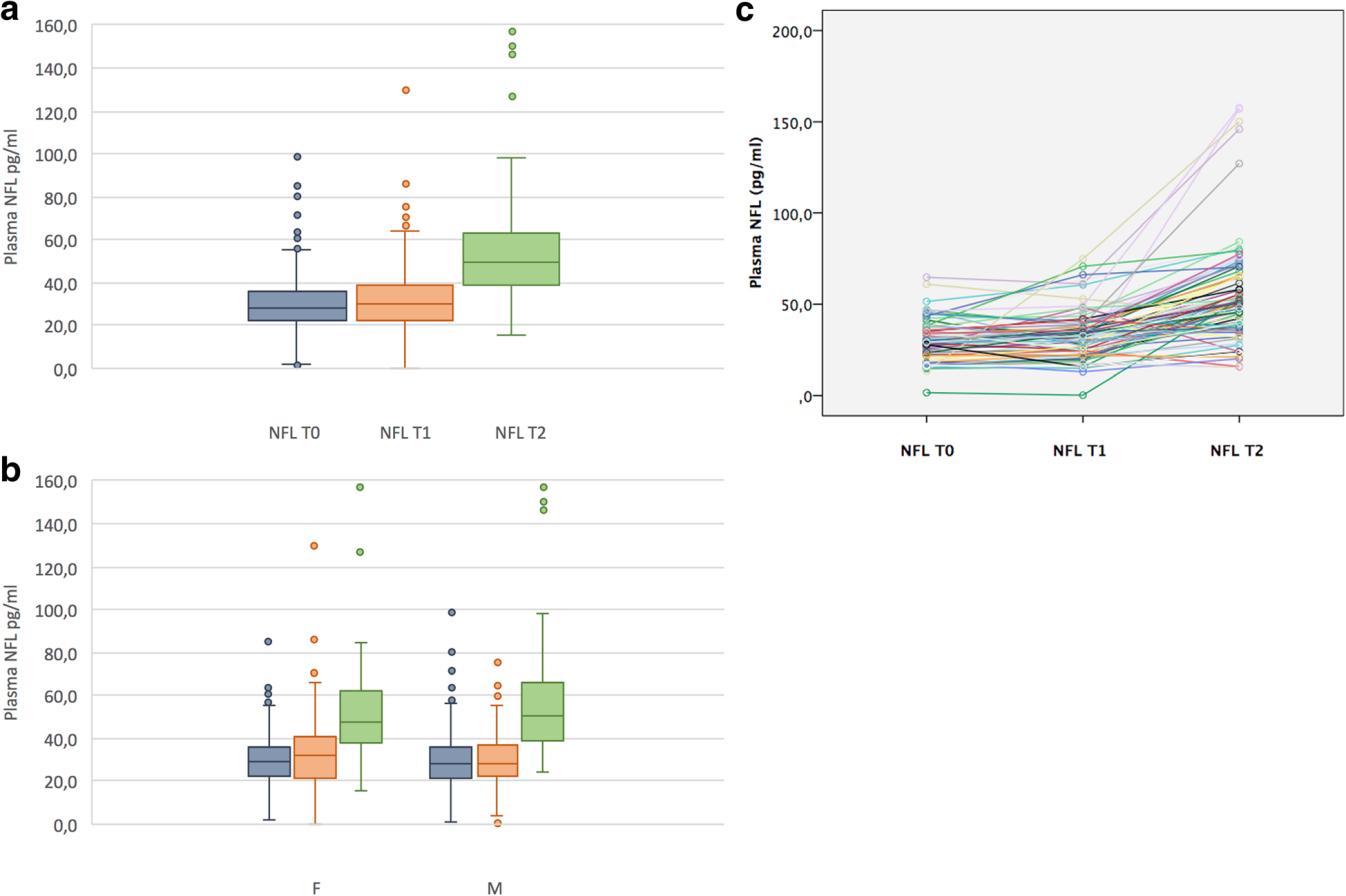
Plasma NFL changes overtime. The first graph (**a**) showed repeated measurements at baseline (T0), 1-year follow-up (T1), and 3-year follow-up (T2). The second one (**b**) charted differences for plasma NFL concentrations stratified by sex at the three time points. The third graph (**c**) reported within person plasma NFL trajectories. Data are referred to a subset of SMC (*N* = 79) who performed T0, T1, and T2 blood samples. Abbreviations: F, female; M, male; NFL, neurofilament light chain; SMC, subjective memory complainers; t-Tau, total Tau; T0, baseline; T1, 1-year follow-up; T2, 3-year follow-up

**Fig. 2:**
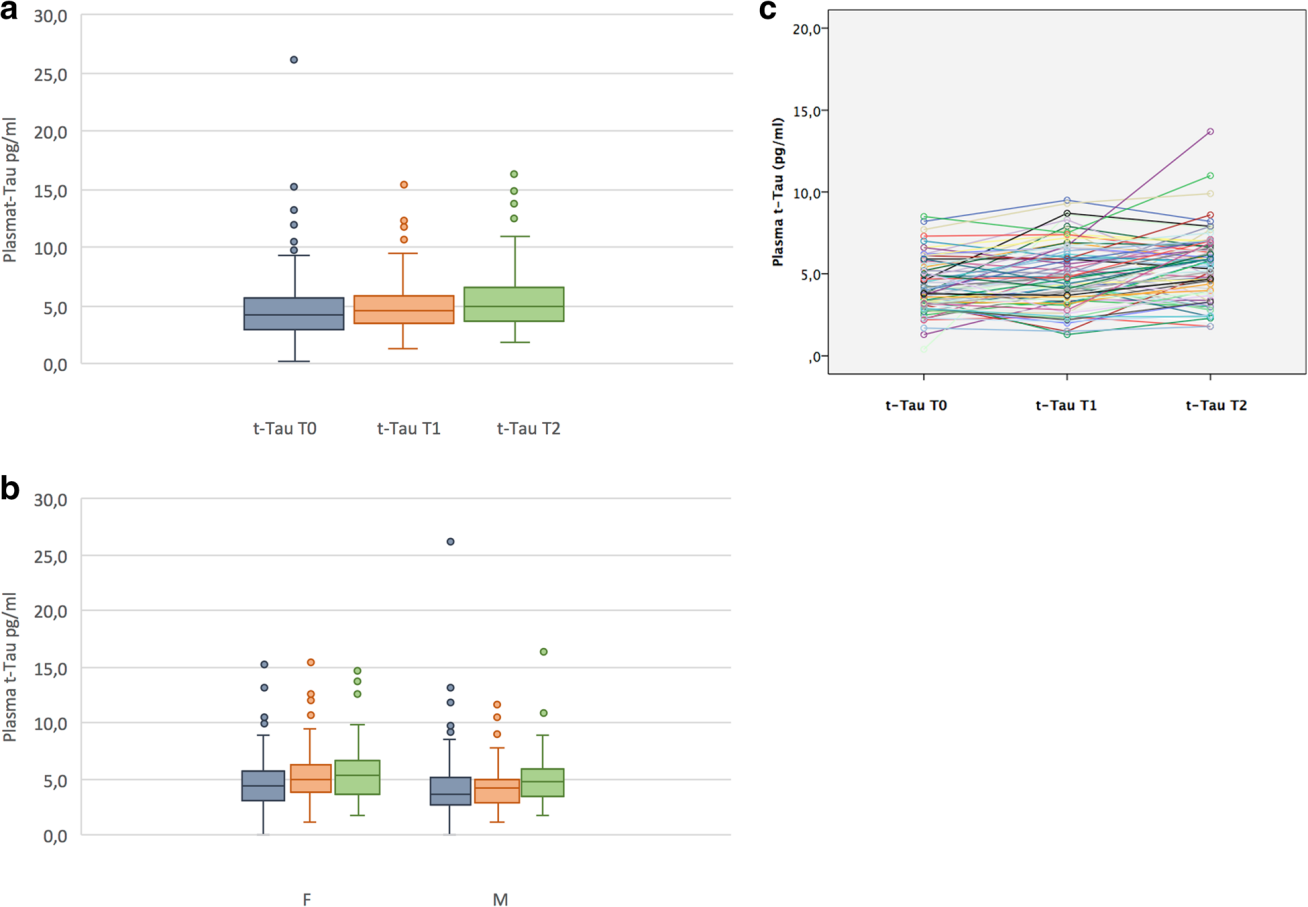
Plasma t-Tau changes overtime. The first graph (**a**) showed repeated measurements at baseline (T0), 1-year follow-up (T1), and 3-year follow-up (T2). The second one (**b**) charted differences for plasma t-Tau concentrations stratified by sex at the three time points. The third graph (**c**) reported within person plasma t-Tau trajectories. Data are referred to a subset of SMC (*N* = 79) who performed T0, T1, and T2 blood samples. Abbreviations: F, female; M, male; NFL, neurofilament light chain; SMC, subjective memory complainers; t-Tau, total Tau; T0, baseline; T1, 1-year follow-up; T2, 3-year follow-up

The clinical and demographic baseline characteristics of the 316 participants also stratified by sex are reported in Table 1. Seventy-nine individuals had all time point plasma samples (at baseline, 1-year, and 3-year follow-up). In these individuals, NFL and t-Tau longitudinal data (Figs. 1a and 2a), also stratified by sex (Figs. 1b and 2b), using within person trajectories (Figs. 1c and 2c), are shown. After a 3-year follow-up, six SMC individuals (~ 2%) clinically progressed to mild cognitive impairment (MCI) or dementia (Table 1S).

**Table 1:** Participant demographics and clinical characteristics at baseline

	SMC tot. (***n*** = 316)	SMC females (***n*** = 200)	SMC males (***n*** = 116)	***p***
Age, mean (SD)	76.1 (3.5)	76.1 (3.3)	76.1 (3.9)	NS
Plasma NFL, mean (SD), pg/mL	30.0 (13.0)	29.9 (11.8)	30.2 (14.8)	NS
Plasma t-Tau, mean (SD), pg/mL	4.5 (2.6)	4.7 (2.4)	4.3 (2.9)	NS
Amyloid PET SUVR, mean (SD)	0.7 (0.1)	0.7 (0.1)	0.7 (0.1)	NS
APOE ɛ4−, No. (%)	254 (80)	159 (79)	95 (82)	
APOE ɛ4+, No. (%)	62 (20)	41 (21)	21 (18)	
Cognitive score				
MMSE, mean (SD)	28.7 (1.0)	28.7 (1.0)	28.6 (1.0)	NS
FCSRT, mean (SD)	46.1 (2.0)	46.1 (2.0)	45.4 (2.1)	NS
Clinical characteristics				
AF, No. (%)	28 (9)	16 (8)	12 (10)	NS
BMI, mean (SD), Kg/m^2^	25.2 (3.5)	25.1 (3.8)	25.5 (2.9)	NS
Diabetes, No. (%)	15 (5)	9 (4.5)	6 (5)	NS
Dyslipidemia, No. (%)	133 (42)	80 (40)	53 (45)	NS
Head trauma, No. (%)	26 (8)	20 (10)	6 (5)	NS
HTA, No. (%)	129 (41)	71 (35)	58 (50)	**0.013**
IHD, No. (%)	35 (11)	17 (8.5)	18 (15)	**0.050**
Mood disorders, No. (%)	85 (27)	71 (35)	14 (12)	**< 0.001**
OSAS, No. (%)	19 (6)	8 (4)	11 (9)	**0.049**
Plasma Vit. B12 deficiency, No. (%)	6 (1.5)	4 (1.5)	2 (1)	NS

*AF* atrial fibrillation, *BMI* body mass index, *FCSRT* free and cued selective reminding test, *HTA* arterial hypertension, *IHD* ischemic heart disease, *MMSE* Mini-Mental State Examination, *NFL* neurofilament light chain, *OSAS* obstructive sleep apnea syndrome, *SD* standard deviation, *SMC* subjective memory complaints, *SUVR* standardized uptake value ratio, *t-Tau* total Tau

### Plasma NFL, t-Tau biomarkers and biological variables

#### The impact of biological factors on plasma biomarkers concentration over time

Table 2 summarizes the main effects investigated for NFL and t-Tau, respectively. For the whole sample, we found a significant effect of age on plasma NFL concentrations (*P* <  0.001), but not of sex and of age*sex interaction. When we performed a post hoc analysis, after dividing the sample into groups of less than and above 76 years of age (value corresponding to average, median and mode), the individuals over 76 years of age showed higher NFL values in all time points considered (mean and SE at baseline: 32.5 ± 1.1 and 27.0 ± 0.9 pg/mL, respectively, *P* <  0.001; at 1-year follow-up: 34.8 ± 1.4 and 27.6 ± 1.2 pg/mL, respectively, *P* <  0.001; at 3-year follow-up: 58.2 ± 3.5 and 49.9 ± 2.7 pg/mL, respectively, *P* = 0.049). When we repeated the analysis, after stratifying by cerebral amyloid-PET-negative and amyloid-PET-positive individuals, we have obtained comparable results. In the amyloid-PET-negative subgroup, the post hoc analysis, after dividing the sample into subsets of less than and above 76 years of age, the individuals over 76 years of age showed higher NFL values in all time points considered (mean and SE at baseline: 31.3 ± 1.3 and 26.2 ± 1.0 pg/mL, respectively, *P* = 0.002; at 1 year: 33.6 ± 1.8 and 28.1 ± 1.5 pg/mL, respectively, *P* = 0.019; at 3 years: 53.8 ± 2.5 and 47.2 ± 2.3 pg/mL, respectively, *P* = 0.050). Again, in amyloid-PET-positive individuals, those over 76 years of age reported higher NFL values (mean and SE at baseline: 35.1 ± 2.1 and 30.4 ± 2.4 pg/mL, respectively, *P* = 0.050; at 1 year: 38.0 ± 2.6 and 27.3 ± 2.7 pg/mL, respectively, *P* = 0.006; at 3 years: 67.8 ± 3.9 and 57.6 ± 3.9 pg/mL, respectively, *P* = 0.049).

**Table 2:** Summary of effect of biological variables and their interactions on longitudinal plasma NFL and t-Tau

	NFL^a^	Sample	NFL^a^	Amyloid- group	NFL^a^	Amyloid+ group
Model	*F* value	*P* value	*F* value	*P* value	*F* value	*P* value
Sex	1.910	0.168	1.482	0.224	0.130	0.719
Age	19.071	**< 0.001**	2.382	**0.002**	2.765	**0.001**
APOE	0.071	0.790	1.067	0.379	2.569	**0.041**
Sex * Age	0.973	0.483	1.622	0.088	1.532	0.178
Sex * APOE	1.375	0.242	0.856	0.464	1.096	0.353
Age * APOE	0.930	0.526	1.367	0.191	1.326	0.195
	t-Tau^b^	Sample	t-Tau^b^	Amyloid− group	t-Tau^b^	Amyloid+ group
Model	*F* value	*P* value	*F* value	*P* value	*F* value	*P* value
Sex	11.639	**0.003**	5.327	**0.022**	2.507	**0.002**
Age	2.320	0.105	1.472	0.186	1.651	0.090
APOE	0.286	0.593	1.598	0.178	1.022	0.399
Sex * Age	4.654	**0.038**	2.317	**0.005**	3.507	**0.030**
Sex * APOE	0.039	0.844	0.877	0.417	0.790	0.501
Age * APOE	0.802	0.667	1.452	0.085	1.550	0.210

^a^Dependent variable: NFL (pg/mL)

^b^Dependent variable: t-Tau (pg/mL)

*APOE ε4* allele had no effect on the total sample and amyloid-PET-negative individuals. However, on amyloid-PET-positive individuals, at 3 years of follow-up, NFL was significantly higher in ε4+ individuals compared to ε4- (mean and SE: 64.1 ± 7.3 and 46.2 ± 3.3 pg/mL, respectively, *P* = 0.040). No interaction with *APOE ε4* carrier status was found in any group.

In the total sample, a significant effect of sex on plasma t-Tau concentrations was reported, displaying higher means in female than in male individuals (*P* = 0.001) (Table 2). The significant sex*age interaction (*P* = 0.038) indicated that this effect was not the same among the different ages. In particular, at 3 years of follow-up, t-Tau concentrations were higher in male individuals aged more than 76 years than in those younger (mean and SE = 6.2 ± 0.8 and 4.5 ± 0.3 pg/mL, respectively, *P* = 0.040) but not in women (mean and SE = 5.2 ± 0.3 and 5.7 ± 0.4 pg/mL, respectively, *P* = 0.359). No significant differences in male or female individuals for baseline and 1 year of follow-up have been observed. When we repeated the analysis, after stratifying participants in cerebral amyloid PET negative and positive, we found similar results in both subgroups, with no significant difference for the baseline and at 1 year of follow-up. Regarding the 3-year follow-up, for amyloid positive individuals, t-Tau concentrations were higher in males aged more than 76 years than in those younger (mean and SE: 7.6 ± 2.1 and 5.5 ± 1.9 pg/mL, respectively, *P* = 0.048), but not in women (mean and SE = 4.9 ± 0.6 and 6.4 ± 1.2 pg/mL, respectively, *P* = 0.220). We found similar results in amyloid-PET-negative individuals: higher concentrations in men aged more than 76 years than in those younger (mean and SE: 5.5 ± 0.6 and 4.0 ± 0.3 pg/mL, respectively, *P* = 0.032) and not in women (mean and SE: 5.5 ± 0.4 and 5.4 ± 0.3 pg/mL, respectively, *P* = 0.886). We did not detect a significant effect of *APOE ε4* allele on plasma t-Tau concentrations nor a significant interaction between *APOE ε4* allele and sex, nor between *APOE ε4* allele and age, both in the total sample and after splitting whole sample in amyloid-PET-negative and positive individuals.

#### Biological factors contributing to high baseline plasma biomarker concentrations

Using baseline NFL as a dependent variable, PLS identified the following variables separating the sample on the basis of NFL concentrations above or below the third quartile: age (VIPs = 2.74) and plasma baseline t-Tau (VIPs = 1.53). The participants with NFL concentration above the third quartile are older and had higher t-Tau concentrations than the others (Table 3). In the second PLS model, the parameters identifying individuals with t-Tau = 0/1 were plasma vitamin B12 deficiency (VIPs = 2.48) and sex (VIPs = 1.83). Individuals with plasma t-Tau concentrations above the third quartile (t-Tau = 1) were more significantly represented by women than men (females = 76%, males = 24%). Individuals with plasma vitamin B12 deficiency showed plasma t-Tau concentrations above the upper quartile (Table 3).

**Table 3:** Partial least square regression models

Index	***NFL VIPs***^***a***^	***t-TAU VIPs***^***a***^
Sex	0.01	1.83
Age	2.74	0.18
t-TAU	1.53	
NFL		0.82
Amyloid PET SUVR	0.69	0.08
APOE ɛ4	0.14	0.53
Cognitive score		
MMSE	1.47	0.72
FCSRT	0.94	0.62
Clinical characteristics		
AF	0.77	0.29
BMI	1.25	0.81
Diabetes	0.38	0.56
Dyslipidemia	0.06	0.34
Head trauma	0.20	0.53
HTA	1.27	0.22
IHD	1.21	0.47
Mood Disorders	0.01	1.19
OSAS	0.80	1.04
Plasma Vit. B12 deficiency	0.01	2.48

*AF* atrial fibrillation, *BMI* body mass index, *FCSRT* free and cued selective reminding test, *HTA* arterial hypertension, *IHD* ischemic heart disease, *MMSE* Mini-Mental State Examination, *NFL* neurofilament light chain, *OSAS* obstructive sleep apnea syndrome, *SD* standard deviation, *SMC* subjective memory complaints, *SUVR* standardized uptake value ratio, *t-Tau* total Tau, *VIPs* variable importance for the projection

^a^VIPs is considered significant when ≥ 1.5

#### Plasma biomarkers and cerebral amyloid load

No association between baseline plasma NFL or t-Tau concentrations and both baseline and 2-year follow-up cerebral Aβ deposition were reported. Baseline plasma NFL concentrations were weakly associated with global brain amyloid increase over time (Δ SUVR, ρs = 0.15; *P* = 0.030). When a subsequent stepwise forward regression using baseline plasma NFL as a dependent variable was performed, a relationship with Δ amyloid deposition in the left posterior cingulate and in the bilateral inferior parietal gyri has been observed (*r* = 0.13; *P* = 0.019 and *r* = 0.14; *P* = 0.013, respectively). Furthermore, baseline plasma NFL concentration was associated with plasma t-Tau (ρs = 0.18; *P* = 0.011) and CSF NFL (ρs = 0.90; *P* <  0.001). In Table 4, we charted a summary of the associations between plasma biomarkers and cerebral amyloid load.

**Table 4:** Correlations between baseline plasma biomarkers and other variables in the SMC population

	Baseline plasma NFL (SMC = 316)		Baseline plasma t-Tau (SMC = 316)	
	ρs	***P*** value	ρs	***P*** value
Baseline				
Amyloid-PET				
SUVR	0.089	0.114	− 0.035	0.535
Age	0.270	< 0.001	− 0.051	0.363
FCSRT	− 0.031	0.585	− 0.031	0.584
MMSE	− 0.059	0.295	0.019	0.742
Plasma NFL			0.180	0.011
Plasma T-Tau	0.180	0.011		
CSF NFL^a^	0.900	< 0.001	− 0.026	0.337
CSF T-Tau^a^	0.540	0.014	− 0.067	0.514
Follow-up				
Amyloid-PET^b^				
SUVR	0.074	0.229	0.016	0.797
Δ SUVR	0.150	0.030	0.037	0.514
FCSRT^c^	− 0.029	0.881	− 0.084	0.173
MMSE^c^	− 0.130	0.029	0.044	0.477
Δ FCSRT^c^	− 0.017	0.787	0.013	0.837
Δ MMSE^c^	0.039	0.526	− 0.083	0.173

*AF* atrial fibrillation, *BMI* body mass index, *FCSRT* free and cued selective reminding test, *HTA* arterial hypertension, *IHD* ischemic heart disease, *MMSE* Mini-Mental State Examination, *NFL* neurofilament light chain, *OSAS* obstructive sleep apnea syndrome, *SD* standard deviation, *SMC* subjective memory complaints, *SUVR* standardized uptake value ratio, *t-Tau* total Tau

^a^Referred to a subsample of 40 SMC

^b^2-year follow-up

^c^3-year follow-up

#### Plasma biomarkers and cognition

No association between baseline plasma NFL or t-Tau concentrations and baseline MMSE score or FCSRT was observed. Baseline plasma NFL concentrations negatively correlated with MMSE at 3-year follow-up (ρs = − 0.13; *P* = 0.029). No association with FCSRT was observed. Δ t-Tau correlated inversely with MMSE (ρs = − 0.19; *P* = 0.015) and positively with Δ NFL (ρs = 0.30; *P* <  0.001) at the 3-year follow-up. In Table 4, we reported a summary of the associations between plasma biomarkers and MMSE and FCSRT scores.

## Discussion

We performed a study on both plasma t-Tau and NFL concentrations longitudinally assessed in a monocentric cohort of cognitively normal individuals with SMC. We found that both plasma t-Tau and NFL concentrations increased over time in our preclinical population. Women had a higher increase of plasma t-Tau concentrations over time compared to men. A positive interaction between female sex and aging to determine a longitudinal increase of t-Tau was also observed. To our knowledge, no other studies reported a significant association between plasma t-Tau concentrations and sex though a higher concentration of tau in CSF of females with AD was reported compared to men [36]. This may depend on differences in sex hormones with a suggested protective role of testosterone against the hyperphosphorylation of tau [36]. Indeed, it is well-known that AD is more prevalent in females than males [37]. We hypothesize that, in our sample, females with SMC showing increased plasma t-Tau concentrations might more probably progress to AD compared to males. These findings indicated that sex-specific reference values may be considered for plasma t-Tau biomarker. By contrast, we showed that sex did not impact plasma NFL concentrations. Indeed, Mattsson and colleagues did not report any sex association in a large cohort including prodromal and AD participants, while two other studies showed an association with men and women, respectively [11, 38]. Although only in CSF, NFL concentrations were higher in men than women in a large meta-analysis of healthy controls and several neurodegenerative diseases including AD [39]. Finally, the association between plasma NFL concentrations and sex remained uncertain and should be elucidated by further longitudinal studies [3, 10].

Age impacted plasma NFL concentrations over time and was associated with plasma NFL. This finding confirms the well-known relationship between aging and NFL [5, 40, 41], though not reported in some diagnostic categories (e.g., multiple sclerosis and Creutzfeldt Jacob disease) [39, 41]. Aging and relative subclinical cerebrovascular alterations per se convey to a subtle neuronal damage and, consequently, the release of axonal byproducts, such as NFL, into body fluids [29, 41, 42]. The relationship between plasma NFL increase and age should be clarified in future studies. Finally, we did not exclude that a high impact of age on plasma NFL concentrations might mask a potential sex effect in our relatively small size cohort. By contrast, plasma t-Tau does not seem to be strictly related to age. However, the positive interaction between female sex and aging, which indicates a longitudinal increase of t-Tau in males, should be further investigated.

Results obtained for SMC individuals did not suggest the presence of higher concentrations of both plasma NFL and t-Tau in *APOE ɛ4* carriers than in non-carriers. This finding is in line with previous studies that did not indicate significant differences between *APOE ɛ4* carriers and non-carriers in terms of plasma NFL concentrations [5, 40]. On the other hand, no association of plasma t-Tau with *APOE ε4* carrier status assessment was shown [3].

The PLS analysis confirmed that age was associated with baseline plasma NFL, whereas female sex with baseline plasma t-Tau concentrations. Additionally, plasma vitamin B12 deficiency was related to baseline plasma t-Tau concentrations. This last finding is of particular clinical relevance and further studies are needed to investigate the potential interaction of t-Tau and vitamin B12 concentrations in blood. A further independent study may check this finding out verifying if this result holds true. In case, a trial with vitamin B12 dietary supplement may be proposed for preclinical individuals with SMC followed with serial plasma t-Tau measurements.

Plasma NFL concentrations weakly correlated with cortical amyloid load deposition at baseline. Plasma NFL was also associated to increased cortical amyloid deposition, at 2-year follow-up, in the left posterior cingulate cortex and bilateral inferior parietal cortex. Interestingly, these areas are part of the default mode network that is functionally impaired in AD [43, 44]. Our analysis indicated an effect of high baseline plasma NFL concentrations on the deposition of amyloid plaques in the brain in a population with SMC. In addition, NFL was significantly higher in amyloid-PET-positive APOE ε4+ allele carriers compared to the ε4− ones at 3 years of follow-up. However, our analysis did not distinguish SMC converters to MCI or dementia from non-converters. In a previous investigation on SMC individuals, only a trend of higher plasma NFL concentrations was described in amyloid-PET-positive SMC participants versus amyloid-PET-negative ones [16]. Other studies evaluating the more advanced prodromal and dementia stages of AD did not demonstrate any association between cerebral amyloid load and plasma NFL concentrations [3, 45]. Finally, the relationship between plasma NFL concentrations and cortical amyloid deposition should be further elucidated.

We demonstrated a cross-sectional and longitudinal association between plasma NFL and t-Tau concentrations. Moreover, the rate of increase of the two plasma biomarkers was related. There is only one previous longitudinal study reporting an association between plasma NFL and t-Tau in a pooled cohort of AD, MCI, and cognitively healthy controls [3]. This suggests that both biomarkers may reflect common neurodegenerative mechanisms in preclinical individuals with SMC.

In line with other studies [5], we found a strong correlation between plasma and CSF NFL concentrations in a subset sample of our SMC individuals [5]. The strict association between plasma and CSF NFL concentrations suggests that NFL alterations in peripheral fluids mirror those occurring in the central nervous system [46].

Baseline plasma NFL concentrations were associated with a 3-year follow-up decline of MMSE score. Our findings are partially in line with those of other studies on prodromal AD and AD dementia [3, 10, 11, 45, 47, 48] and with a study on preclinical AD individuals [16]. The rate of change of t-Tau, but not of NFL, was associated with a decrease in MMSE score at three-year follow-up. The use of serial plasma biomarker measurements and the rate of change of concentrations over time may be more reliable than absolute values, at a single time point, in differentiating patients with neurodegenerative diseases from controls [47]. In this regard, a recent study confirmed that the rate plasma t-Tau increase may be predictive of the dementia development and be adopted as a biomarker for risk stratification [13].

Finally, the lack of associations of NFL and t-Tau with a decrease in FCSRT score over time here reported is possibly due to the relative short length of follow-up and the low rate of conversion in our SMC population (*N* = 6 persons, 2%). Therefore, our results on cognitive decline should be cautiously considered as a preliminary.

### Limitations of study

Our study presents some caveats. First, the low number of converters (SMC-MCI and SMC-dementia) does not allow any definitive conclusions on the role of plasma NFL and t-Tau in predicting cognitive worsening over time in preclinical individuals with SMC. Furthermore, we miss some sample collection along the time points, and we lack imaging data regarding brain metabolism and atrophy. Potential effects of genetic polymorphisms other than *APOE ε4* allele on modifications of plasma NFL and t-Tau concentrations could not be ruled out. The t-Tau assay does not have a good linearity and parallelism and our results need to be confirmed in further investigations.

## Conclusion and future direction

We reported significantly increased concentrations of plasma t-Tau in female sex and the potential association of plasma vitamin B12 deficiency with plasma t-Tau concentrations. We confirmed the significant association of plasma NFL concentrations with age and the correlation with its CSF concentrations. It was a potential predictor of longitudinal cerebral amyloid deposition in specific brain areas associated with AD pathophysiology. Rate of changes of plasma t-Tau may predict longitudinal worsening of cognition in SMC individuals. We also supported previous results reporting no association between plasma NFL and t-Tau with *APOE ε4* allele. In this regard, age, sex, and *APOE ε4* allele combined with plasma NFL and t-Tau may be used to establish a risk stratification score integrating different information on the development of dementia.

In light of our findings, we believe that the impact of biological variables needs to be critically assessed in the biomarker discovery and development process for AD diagnosis [49]. Moreover, our results should not be generalizable to other more specific settings (e.g., clinical population studies), different geographies, and ethnic groups.

Exploring the impact of these biological variables on the concentrations and longitudinal trajectories of plasma NFL and t-Tau in cognitively normal individuals, independently of their stability or their development of cognitive decline over time, is crucial to define the potential role of plasma NFL and t-Tau as diagnostic, prognostic, and theragnostic biomarkers. Importantly, our study also suggested that NFL and t-Tau seem not to be considerably influenced by comorbidities and vascular risk factors and remain relatively stable. Indeed, these steps will allow to better delineate their potential context-of-use for different settings, including clinical practice and pharmacological trials. In this respect, the recruitment of individuals for clinical trials will be improved using pathophysiological blood-based biomarkers detecting individuals at risk for progression and decline. We foresee the option to enter a novel era of next-generation biomarker-guided targeted therapies for AD and other neurodegenerative diseases under the paradigm of precision medicine [50].

## Supplementary Information

**Additional file 1:** **Table 1S.** Demographic, clinical and biomarkers description of the progressive SMC (*N* = 6) at follow-up. Abbreviations: MMSE, Mini-Mental State Examination score; FCSRT, Free and Cued Selective Rating Test; NFL, neurofilament light chain; t-Tau, total Tau.

## Data Availability

The dataset generated and analyzed in the current study is available from the corresponding author on reasonable request.

## Abbreviations

AD: Alzheimer’s disease
APMI: Alzheimer Precision Medicine Initiative
Aβ_1–42_: Amyloid-β 1 to 42
ANOVA: Analysis of variance
HTA: Arterial hyperthension
BMI: Body mass index
CSF: Cerebrospinal fluid
FCSRT: Free and Cued Selective Rating Test
INSIGHT-preAD: INveStIGation of AlzHeimer’s PredicTors in Subjective Memory Complainers”
LMM: Linear mixed models
MCI: Mild cognitive impairment
MMSE: Mini-Mental State Examination
OSAS: Obstructive sleep apnea syndrome
NFL: Neurofilament light chain
PLS: Two partial least square
SD: Standard deviation
SMC: Subjective memory complaint
SUVR: Standard uptake value ratios
t-Tau: Total Tau
VIPs: Variable Importance in Projection

## References

[1] Molinuevo JL, Ayton S, Batrla R, Bednar MM, Bittner T, Cummings J (2018). Current state of Alzheimer’s fluid biomarkers. Acta Neuropathol (Berl).

[2] Mattsson N, Zetterberg H, Janelidze S, Insel PS, Andreasson U, Stomrud E (2016). Plasma tau in Alzheimer disease. Neurology.

[3] Mattsson N, Andreasson U, Zetterberg H, Blennow K (2017). Alzheimer’s disease neuroimaging initiative. Association of plasma neurofilament light with neurodegeneration in patients with Alzheimer disease. JAMA Neurol.

[4] Hampel H, O’Bryant SE, Molinuevo JL, Zetterberg H, Masters CL, Lista S, et al. Blood-based biomarkers for Alzheimer disease: mapping the road to the clinic. Nat Rev Neurol. 2018. 10.1038/s41582-018-0079-7. 10.1038/s41582-018-0079-7 PMC6211654 30297701

[5] Khalil M, Teunissen CE, Otto M, Piehl F, Sormani MP, Gattringer T (2018). Neurofilaments as biomarkers in neurological disorders. Nat Rev Neurol.

[6] Lue L-F, Sabbagh MN, Chiu M-J, Jing N, Snyder NL, Schmitz C (2017). Plasma levels of Aβ42 and tau identified probable Alzheimer’s dementia: findings in two cohorts. Front Aging Neurosci.

[7] Xia J, Broadhurst DI, Wilson M, Wishart DS (2013). Translational biomarker discovery in clinical metabolomics: an introductory tutorial. Metabolomics Off J Metabolomic Soc.

[8] Kinnunen KM, Greenwood R, Powell JH, Leech R, Hawkins PC, Bonnelle V (2011). White matter damage and cognitive impairment after traumatic brain injury. Brain J Neurol.

[9] Shahim P, Tegner Y, Gustafsson B, Gren M, Ärlig J, Olsson M (2016). Neurochemical aftermath of repetitive mild traumatic brain injury. JAMA Neurol.

[10] Zhou W, Zhang J, Ye F, Xu G, Su H, Su Y (2017). Plasma neurofilament light chain levels in Alzheimer’s disease. Neurosci Lett.

[11] Lin Y-S, Lee W-J, Wang S-J, Fuh J-L (2018). Levels of plasma neurofilament light chain and cognitive function in patients with Alzheimer or Parkinson disease. Sci Rep.

[12] Mattsson N, Cullen NC, Andreasson U, Zetterberg H, Blennow K. Association between longitudinal plasma neurofilament light and neurodegeneration in patients with Alzheimer disease. JAMA Neurol. 2019. 10.1001/jamaneurol.2019.0765. 10.1001/jamaneurol.2019.0765 PMC6583067 31009028

[13] Pase MP, Beiser AS, Himali JJ, Satizabal CL, Aparicio HJ, DeCarli C, et al. Assessment of plasma total tau level as a predictive biomarker for dementia and related endophenotypes. JAMA Neurol. 2019. 10.1001/jamaneurol.2018.4666. 10.1001/jamaneurol.2018.4666 PMC6515589 30830207

[14] Dage JL, Wennberg AMV, Airey DC, Hagen CE, Knopman DS, Machulda MM (2016). Levels of tau protein in plasma are associated with neurodegeneration and cognitive function in a population-based elderly cohort. Alzheimers Dement J Alzheimers Assoc.

[15] Park J-C, Han S-H, Yi D, Byun MS, Lee JH, Jang S, et al. Plasma tau/amyloid-β1-42 ratio predicts brain tau deposition and neurodegeneration in Alzheimer’s disease. Brain J Neurol. 2019. 10.1093/brain/awy347. 10.1093/brain/awy347 30668647

[16] Chatterjee P, Goozee K, Sohrabi HR, Shen K, Shah T, Asih PR (2018). Association of plasma neurofilament light chain with neocortical amyloid-β load and cognitive performance in cognitively normal elderly participants. J Alzheimers Dis JAD.

[17] Mielke MM, Hagen CE, Wennberg AMV, Airey DC, Savica R, Knopman DS (2017). Association of plasma total tau Level with cognitive decline and risk of mild cognitive impairment or dementia in the Mayo Clinic study on aging. JAMA Neurol.

[18] Müller S, Preische O, Göpfert JC, Yañez VAC, Boecker H, Joos TO (2017). Tau plasma levels in subjective cognitive decline: results from the DELCODE study. Sci Rep.

[19] Dubois B, Epelbaum S, Nyasse F, Bakardjian H, Gagliardi G, Uspenskaya O (2018). Cognitive and neuroimaging features and brain β-amyloidosis in individuals at risk of Alzheimer’s disease (INSIGHT-preAD): a longitudinal observational study. Lancet Neurol.

[20] Hampel H, O’Bryant SE, Castrillo JI, Ritchie C, Rojkova K, Broich K (2016). PRECISION MEDICINE - the golden gate for detection, treatment and prevention of Alzheimer’s disease. J Prev Alzheimers Dis.

[21] Hampel H, O’Bryant SE, Durrleman S, Younesi E, Rojkova K, Escott-Price V (2017). A precision medicine initiative for Alzheimer’s disease: the road ahead to biomarker-guided integrative disease modeling. Climacteric.

[22] Hampel H, Toschi N, Babiloni C, Baldacci F, Black KL, Bokde ALW (2018). Revolution of Alzheimer precision neurology. Passageway of systems biology and neurophysiology. J Alzheimers Dis JAD.

[23] Hampel H, Vergallo A, Perry G, Lista S. Alzheimer Precision Medicine Initiative (APMI). The Alzheimer Precision Medicine Initiative. J Alzheimers Dis JAD. 2019. 10.3233/JAD-181121. 10.3233/JAD-181121 30814352

[24] Habert M-O, Bertin H, Labit M, Diallo M, Marie S, Martineau K (2018). Evaluation of amyloid status in a cohort of elderly individuals with memory complaints: validation of the method of quantification and determination of positivity thresholds. Ann Nucl Med.

[25] Schwarz CG, Senjem ML, Gunter JL, Tosakulwong N, Weigand SD, Kemp BJ (2017). Optimizing PiB-PET SUVR change-over-time measurement by a large-scale analysis of longitudinal reliability, plausibility, separability, and correlation with MMSE. NeuroImage.

[26] Hampel H, Toschi N, Baldacci F, Zetterberg H, Blennow K, Kilimann I (2018). Alzheimer’s disease biomarker-guided diagnostic workflow using the added value of six combined cerebrospinal fluid candidates: Aβ1-42, total-tau, phosphorylated-tau, NFL, neurogranin, and YKL-40. Alzheimers Dement J Alzheimers Assoc.

[27] Baldacci F, Toschi N, Lista S, Zetterberg H, Blennow K, Kilimann I, et al. Two-level diagnostic classification using cerebrospinal fluid YKL-40 in Alzheimer’s disease. Alzheimers Dement J Alzheimers Assoc. 2017. 10.1016/j.jalz.2017.01.021. 10.1016/j.jalz.2017.01.021 28263742

[28] Olsson B, Lautner R, Andreasson U, Öhrfelt A, Portelius E, Bjerke M, et al. CSF and blood biomarkers for the diagnosis of Alzheimer’s disease: a systematic review and meta-analysis. Lancet Neurol. 2016. 10.1016/S1474-4422(16)00070-3. 10.1016/S1474-4422(16)00070-3 27068280

[29] Ashton NJ, Leuzy A, Lim YM, Troakes C, Hortobágyi T, Höglund K (2019). Increased plasma neurofilament light chain concentration correlates with severity of post-mortem neurofibrillary tangle pathology and neurodegeneration. Acta Neuropathol Commun.

[30] Palmqvist S, Insel PS, Zetterberg H, Blennow K, Brix B, Stomrud E (2019). Accurate risk estimation of β-amyloid positivity to identify prodromal Alzheimer’s disease: cross-validation study of practical algorithms. Alzheimers Dement.

[31] Randall J, Mörtberg E, Provuncher GK (2013). Tau proteins in serum predict neurological outcome after hypoxic brain injury from cardiac arrest: results of a pilot study. Resuscitation.

[32] Gisslén M, Price RW, Andreasson U (2015). Plasma concentration of the Neurofilament light protein (NFL) is a biomarker of CNS injury in HIV infection: a cross-sectional study. EBioMedicine.

[33] Applied Mixed Models in Medicine, 2nd Edition. WileyCom n.d. https://www.wiley.com/en-us/Applied+Mixed+Models+in+Medicine%2C+2nd+Edition-p-9780470023563. Accessed 19 Feb 2019.

[34] Abdi H, Williams LJ (2013). Partial least squares methods: partial least squares correlation and partial least square regression. Methods Mol Biol.

[35] Barker M, Rayens W (2003). Partial least squares for discrimination. J Chemom.

[36] Sundermann EE, Panizzon MS, Chen X (2020). Sex differences in Alzheimer’s-related tau biomarkers and a mediating effect of testosterone. Biol Sex Differ.

[37] Alzheimer Association (2016). 2016 Alzheimer’s disease facts and figures. Alzheimers Dement.

[38] Hansson O, Janelidze S, Hall S, Magdalinou N, Lees AJ, Andreasson U (2017). Blood-based NfL: a biomarker for differential diagnosis of parkinsonian disorder. Neurology.

[39] Bridel C, van Wieringen WN, Zetterberg H (2019). Diagnostic value of cerebrospinal fluid neurofilament light protein in neurology: a systematic review and meta-analysis. JAMA Neurol.

[40] Gaetani L, Blennow K, Calabresi P, Di Filippo M, Parnetti L, Zetterberg H. Neurofilament light chain as a biomarker in neurological disorders. J Neurol Neurosurg Psychiatry. 2019. 10.1136/jnnp-2018-320106. 10.1136/jnnp-2018-320106 30967444

[41] Palermo G, Mazzucchi S, Della Vecchia A, et al. Different clinical contexts of use of blood neurofilament light chain protein in the spectrum of neurodegenerative diseases [published online ahead of print, 2020 Aug 9]. Mol Neurobiol. 2020;10. 10.1007/s12035-020-02035-9. 10.1007/s12035-020-02035-9 32772223

[42] Vågberg M, Norgren N, Dring A, Lindqvist T, Birgander R, Zetterberg H (2015). Levels and age dependency of neurofilament light and glial Fibrillary acidic protein in healthy individuals and their relation to the brain parenchymal fraction. PLoS One.

[43] Greicius MD, Krasnow B, Reiss AL, Menon V (2003). Functional connectivity in the resting brain: a network analysis of the default mode hypothesis. Proc Natl Acad Sci U S A.

[44] Chiesa PA, Cavedo E, Vergallo A, Lista S, Potier M-C, Habert M-O, et al. Differential default mode network trajectories in asymptomatic individuals at risk for Alzheimer’s disease. Alzheimers Dement J Alzheimers Assoc. 2019. 10.1016/j.jalz.2019.03.006. 10.1016/j.jalz.2019.03.006 31113760

[45] Lewczuk P, Ermann N, Andreasson U, Schultheis C, Podhorna J, Spitzer P (2018). Plasma neurofilament light as a potential biomarker of neurodegeneration in Alzheimer’s disease. Alzheimers Res Ther.

[46] Baldacci F, Mazzucchi S, Della Vecchia A, Giampietri L, Giannini N, Koronyo-Hamaoui M (2020). The path to biomarker-based diagnostic criteria for the spectrum of neurodegenerative diseases. Expert Rev Mol Diagn.

[47] Preische O, Schultz SA, Apel A, Kuhle J, Kaeser SA, Barro C, et al. Serum neurofilament dynamics predicts neurodegeneration and clinical progression in presymptomatic Alzheimer’s disease. Nat Med. 2019. 10.1038/s41591-018-0304-3. 10.1038/s41591-018-0304-3 PMC6367005 30664784

[48] Weston PSJ, Poole T, Ryan NS, Nair A, Liang Y, Macpherson K (2017). Serum neurofilament light in familial Alzheimer disease. Neurology.

[49] Baldacci F, Lista S, Vergallo A, Palermo G, Giorgi FS, Hampel H (2019). A frontline defense against neurodegenerative diseases:the development of early disease detection methods. Expert Rev Mol Diagn.

[50] Penner G, Lecocq S, Chopin A, Vedoya X, Lista S, Vergallo A (2019). Blood-based diagnostics of Alzheimer’s disease. Expert Rev Mol Diagn.

