# Supplementary material for: Age and sex impact plasma NFL and t-Tau trajectories in individuals with subjective memory complaints: a 3-year follow-up study
Source: Alzheimers Res Ther. 2020 Nov 12;12:147. doi: 10.1186/s13195-020-00704-4 (PMC7663867; doi:10.1186/s13195-020-00704-4)
Supplement: Supplementary file 1 — Additional file 1: Table 1S. Demographic, clinical and biomarkers description of the progressive SMC (N = 6) at follow-up. Abbreviations: MMSE, Mini-Mental State Examination score; FCSRT, Free and Cued Selective Rating Test; NFL, neurofilament light chain; t-Tau, total Tau. [file 13195_2020_704_MOESM1_ESM.docx]

**Supplementary table**

**Table** **1S**. Demographic, clinical and biomarkers description of the progressive SMC (N = 6) at follow-up.

| **Variable** | **Mean** ± **SD** | |
| --- | --- | --- |
| Sex (M / F) | 4 / 2 |  |
| Age | 79.2 ± 4.1 years |  |
| Baseline plasma NFL | 38.9 ± 19.2 pg/mL |  |
| Baseline plasma t-Tau | 5.9 ± 3.8 pg/mL |  |
| 3-year follow-up plasma NFL | 34.6 ± 16.4 pg/mL |  |
| 3-year follow-up plasma t-Tau | 6.1 ± 3.0 pg/mL |  |
| Baseline MMSE | 28.3 ± 0.5 |  |
| Baseline FCSRT | 45.3 ± 1.5 |  |
| 3-year follow-up MMSE | 24.3 ± 5.7 |  |
| 3-year follow-up FCSRT | 34.3 ± 10.0 |  |

*Abbreviations:*

MMSE, Mini-Mental State Examination score; FCSRT, Free and Cued Selective Rating Test; NFL, neurofilament light chain; t-Tau, total Tau.
